# The Wesleyans and Hospital Sunday

**Published:** 1887-02-05

**Authors:** Benjamin Smith

**Affiliations:** Superintendent Minister. 90, Great King Street, Macclesfield


### THE EDITOR'S LETTER-BOX.

[Our Correspondents are reminded that prolixity is a great bar to publication, and that brevity of style and conciseness of statement greatly facilitate early publication.]

### THE WESLEYANS AND HOSPITAL SUNDAY.

Mr. May's letter published in your issue of the 22nd inst., was pointed out to me to-day. I have read it with surprise and regret. The Wesleyans in Macclesfield have not declined to make collections on behalf of the Infirmary. Each of the three congregations in Macclesfield has made two collections every year. Other Wesleyan congregations in the circuit have done the same. Like some other congregations in this neighbourhood and elsewhere, they have not always thought it advisable to appeal to their friends on the exact day appointed by the Infirmary Committee. But they have cheerfully done their best. You will now be still able to affirm that you know of no case in which Wesleyans decline to render help to this important work. Perhaps you may think it undesirable to proceed further in this matter. But as you have published the error (unwittingly I am sure), I may rely on your publishing the rectification early, and in similar type.

Benjamin Smith,
Superintendent Minister.

90, Great King Street, Macclesfield.

